# GRank: a middleware search engine for ranking genes by relevance to given genes

**DOI:** 10.1186/1471-2105-14-251

**Published:** 2013-08-19

**Authors:** Kamal Taha, Dirar Homouz, Hassan Al Muhairi, Zaid Al Mahmoud

**Affiliations:** 1 Department of Electrical and Computer Engineering, Khalifa University, Abu Dhabi, UAE

## Abstract

**Background:**

Biologists may need to know the set of genes that are *semantically related* to a given set of genes. For instance, a biologist may need to know the set of genes related to another set of genes known to be involved in a specific disease. Some works use the concept of gene *clustering* in order to identify semantically related genes. Others propose tools that return the set of genes that are semantically related to a given set of genes. Most of these gene similarity measures determine the semantic similarities among the genes based solely on the proximity to each other of the GO terms annotating the genes, while overlook the *structural dependencies* among these GO terms, which may lead to low *recall* and *precision* of results.

**Results:**

We propose in this paper a search engine called GRank, which overcomes the limitations of the current gene similarity measures outlined above as follows. It employs the concept of *existence dependency* to determine the *structural dependencies* among the GO terms annotating a given set of gene. After determining the set of genes that are semantically related to input genes, GRank would use microarray experiment to *rank* these genes based on their *degree of relativity* to the input genes. We evaluated GRank experimentally and compared it with a comparable gene prediction tool called DynGO, which retrieves the genes and gene products that are *relatives* of input genes. Results showed marked improvement.

**Conclusions:**

The experimental results demonstrated that GRank overcomes the limitations of current gene similarity measures. We attribute this performance to GRank’s use of *existence dependency* concept for determining the semantic relationships among gene annotations. The *recall* and *precision* values for two benchmarking datasets showed that GRank outperforms DynGO tool, which does not employ the concept of existence dependency. The demo of GRank using 11000 KEGG yeast genes and a **G**ene **E**xpression **O**mnibus (GEO) microarray file named “GSM34635.pad” is available at: http://ecesrvr.kustar.ac.ae:8080/ (click on the link labelled Gene Ontology 2).

## Background

Biologists often need to know the set *S*′ of genes that are *semantically related* to a given set *S* of genes. Determining the set *S*′ helps in understanding gene-disease interactions and advanced disease diagnosis. For instance, biologists in the UAE are trying to determine the set of genes that are related to the genes involved in Type 2 Diabetes (T2D) *(one out of five people in the UAE between the ages of 20 to 79 lives with T2D)*. Some works (e.g., DynGO [1]) propose tools that return the set of genes that are semantically related to a given set of genes. For instance, DynGO “retrieves genes and gene products that are *relatives* of input genes based on similar GO annotations, and displays the related genes and gene products in an association tree” [1,2]. Other works use the concept of gene *clustering* in order to identify semantically related genes [3-5]. However, most of these methods determine the semantic similarities among the genes based solely on the proximity to each other of the GO terms annotating the genes, while overlook the *structural dependencies* among these GO terms, which may lead to low *recall* and *precision* of results.

Most similarity measure approaches can be classified into three: edge-based, node-based, and Hybrid methods. Edge-based measures [6,7] rely on counting edges in the graph. In most of these measures, the shortest path length is used as a distance measure between two terms in a graph. Node-based measures [8-12] exploit the information content (IC) of two terms being compared and of their LCA. If this LCA has high information content, the two terms are considered to be semantically similar. Hybrid methods [13] combine edge-based and node-based methods. Edge-based measures assume that: (1) nodes and edges are uniformly distributed, and (2) edges at the same level in a hierarchy correspond to the same semantic distance between terms. These assumptions are not always true in biological ontologies. As for node-based measures, their limitations are: (1) they do not take into account the *distance* separating GO terms from their LCA [9], (2) they use IC as the major factor for determining the semantic similarity of GO terms, which is inappropriate, (3) some of them rely only on the *number* of common ancestor terms, while overlooking their semantic contributions to the two terms under consideration, and (4) many of these methods overlook the information contained in the structure of the ontology and concentrate only on the information content of a term derived from the corpus statistics. Most of these algorithms determine the semantic similarities on term by term basis and therefore ignore the context of a gene which consists of multiple terms. Thus, such method cannot be easily implemented to infer the functional similarities among different groups of genes.

Nagar et al*.*[14] define the path length (PL) function between two GO terms as the *minimum path length* in the GO graph between the two terms. Chagoyen and Pazos [6] relate the functional coherence of GO terms *GO*_*i*_ and *GO*_*j*_ to the number of proteins in *GO*_*j*_ that are functionally associated with proteins in *GO*_*i*_ and use the cumulative hypergeometric distribution.

## Methods

**Figure 1:**
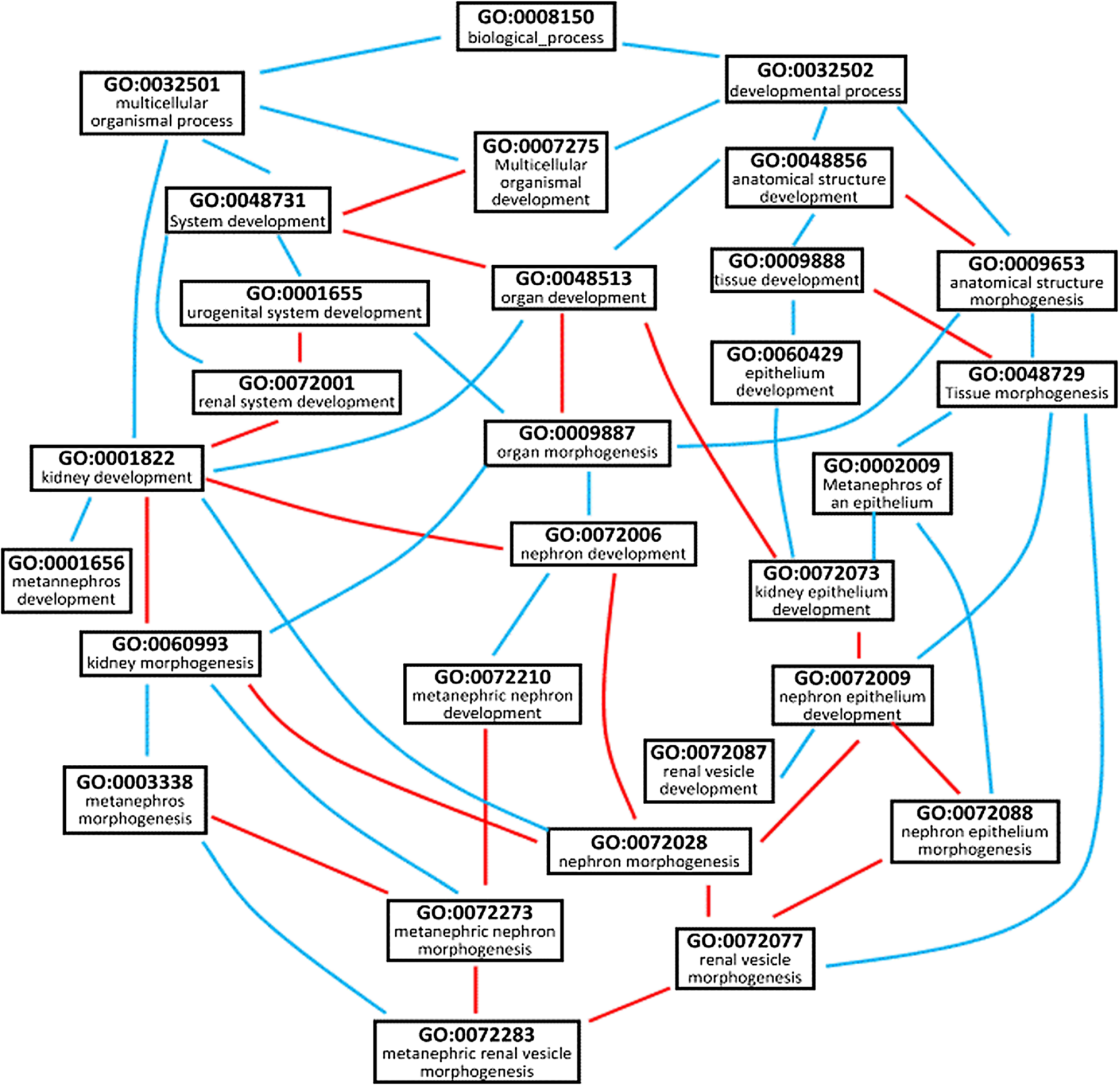
**A fragment of GO Graph showing the ontological relationships of 29 GO terms.***Blue edges denote “is-a” relations and red edges denote “part-of” relations.*

In the framework of GRank, the structure of GO is described in terms of a graph, which we call GO Graph. In this graph, GO terms are nodes and the relationships between the terms are edges. For example, Figure 1 presents a fragment of a GO Graph showing the ontological relationships of 29 GO terms. GRank accepts Keyword-based queries with the form *Q* (*“g*_*1*_”, “*g*_*2*_”, .., “*g*_*n*_”), where *g*_*i*_ denotes a gene (or a gene product) keyword.

**Figure 2:**
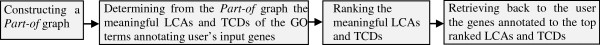
A graphical representation of our approach showing the sequential processing steps for answering a query.

User selects an input *(a query, which is composed of genes annotated to GO)*. GRank would then map these genes to a set of GO terms. Let *S*_*T*_ denote these GO terms. GRank finds the *meaningful Lowest Common Ancestors* (LCA) and the *meaningful Top Common Descendants* (TCD) of the set *S*_*T*_. A *meaningful* LCA is a LCA in GO graph, on which the *existence* of *S*_*T*_ depends. A *meaningful* TCD is a TCD in GO graph, whose existence depends on the set *S*_*T*_. GRank would then rank the meaningful LCAs and TCDs, which are then converted back to genes based on annotations and retrieved back to the user. The genes annotated to the meaningful LCAs and the meaningful TCDs are the most semantically related to the user’s input genes. Figure 2 is an overview of our approach. It shows the sequential processing steps for answering a query. Because there are many abbreviations of concepts in the paper, we summarize them in Table 1.

**Table 1:** **Abbreviations of Concept (*abb *denotes abbreviation)**

***Abb***	***Concept***	***Abb***	***Concept***
**KC**	Keyword Context	**TCD**	Top Common Descendant
**LCA**	Lowest Common Ancestor	***T***∈**SR**_**KC**_	Term *T* is semantically related to the KC
**POG**	Part-Of Graph	**DPT**_**x**_	Depth of GO term x
**SR**_**KC**_	The set of GO terms that are semantically related to the KC	**S( *v *) and R( *v *)**	S(*v*): The score of GO term v. R(*v*): The rank of GO term *v*

### Constructing a graph based on part-of relations

**Figure 3:**
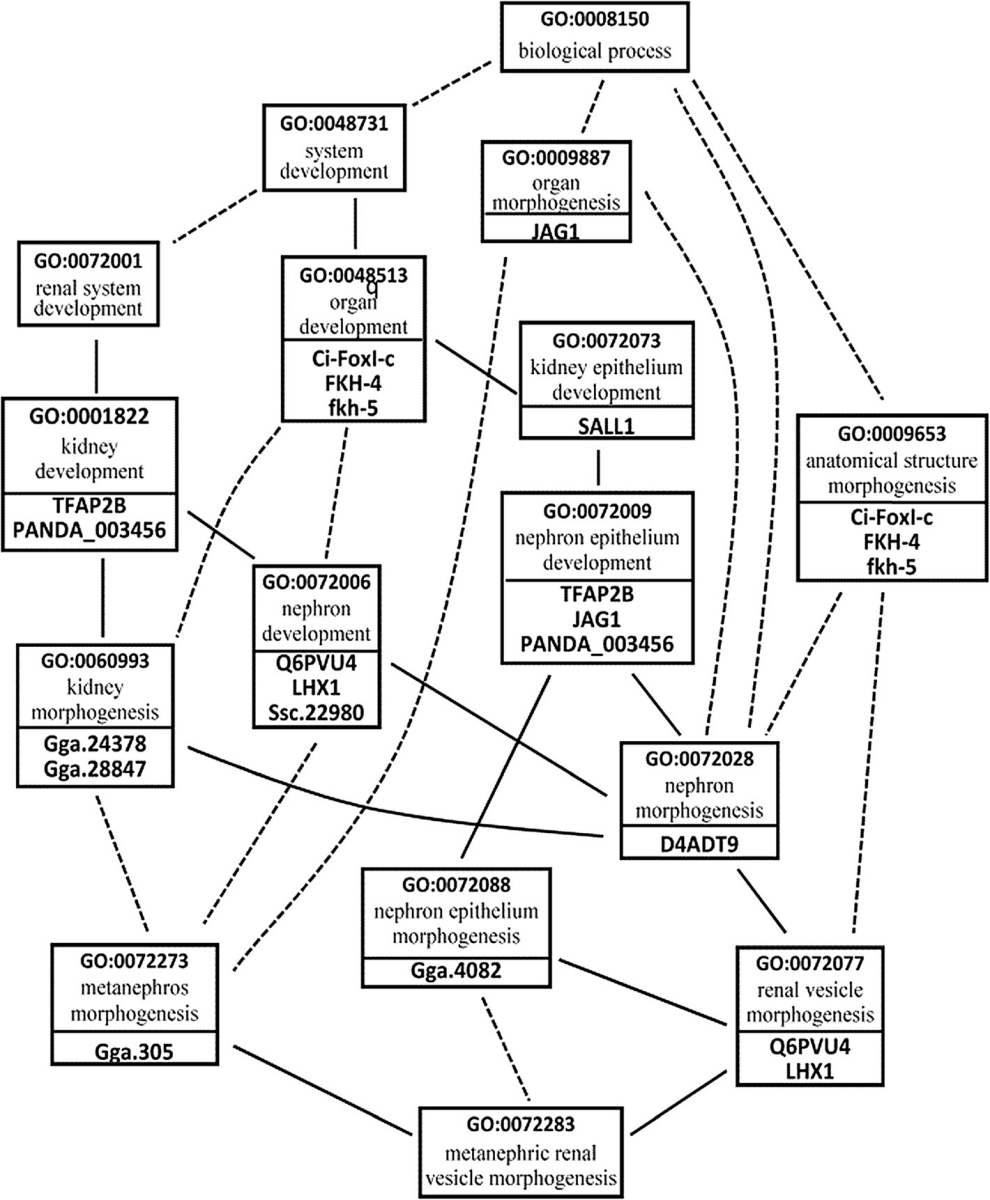
**POG constructed from the GO Graph in Figure**1**.** Dotted lines denote inferred *part-of* relationships and solid lines denote explicit *part-of* relationships. Some relationships are omitted from the figure for the sake of figure clarity. Each term node is accompanied with the genes annotated to it.

*Notation 1, Keyword Context (KC): A KC is a GO term annotated to a query gene product. For example, consider Figures*1*and*3*and the query Q(“JAG1”). The term “organ morphogenesis” (GO:0009887) is a KC because the gene “JAG1” is annotated to it.*

Let *S*_*KC*_ be a set of KCs annotating the user’s input genes. To construct the answer for this query, GRank needs to identify the meaningful LCAs and the meaningful TCDs of the set *S*_*KC*_ based on the concept of existence dependency. Towards this, GRank will need to check all “*part-of”* relations in GO graph, because*: “part of has a specific meaning in GO and a part of relation would only be added between A and B if B is necessarily part of A: wherever B exists, it is as part of A, and the presence of the B implies the presence of A*” [15]. “*part-of relation embodies some aspects of existence dependency. A part-of relation with existence dependent parts can simply be replaced by existence dependency: in case of existence dependent components, the existence dependency relation is identical to the part of relation*” [16]*.*

Since not all “part-of” relations are explicitly expressed in GO Graph (*some can be inferred from the graph*), GRank converts the GO Graph into a graph called Part-Of Graph (POG), which contains only the explicit and inferred “part-of” relations. The LCAs/TCDs of KCs will be determined from the POG and not from the GO Graph. A POG is a GO Graph after: (1) removing all its relations except for the “part-of” ones, and (2) adding the inferred “part-of” relations. The terms *A* and *B* are connected by a “part-of” relation in the POG, if the GO Graph either states this relation expressly or it can be inferred from the graph using the following two *inference rules* described in [15]: (1) if *A* “is-a*” B* and *B* is “part-of*” C*, *A* is “part-of*” C*, and (2) if *A* is “part-of*” B* and *B* “is-a*” C*, *A* is “part-of*” C*.

Figure 3 shows a fragment of POG derived from the GO Graph in Figure 1. For example, since in Figure 1: (1) the term *multicellular organismal process* (GO:0032501) “is-a” the term *biological process* (GO:0008150), (2) the term *multicellular organismal development* (GO:0007275) “is-a” the term *multicellular organismal process* (GO:0032501), and (3) the term *system development* (GO:0048731) is “part-of” the term *multicellular organismal development* (GO:0007275), then in Figure 3 the term *system development* (GO:0048731) is “part-of” the term *biological process* (GO:0008150). In Figure 3, each term node is accompanied with the genes annotated to it.

### Determining the depths of terms

We observe that in order for a LCA/TCD of KCs to be *meaningful*, the terms located in each path from the LCA/TCD to a KC in POG should have *unique depths* based on their “*is-a*” relations in GO graph. “is-a” is a simple type-subtype relation between two GO terms [15]. Consider that: (1) *A*′ “is-a” *A*, (2) *A* “is-a” *C*, (3) *B*′ “is-a” *B*, and (4) *B* “is-a” *C*. Both of the terms *A* and *B* inherit the characteristics and properties of their supertype *C*. Therefore, *A* and *B* share common characteristics. Since *A*′ and *B*′ inherit from the characteristics and properties of terms that have the same depth (*the terms A and B*), *A*′ and *B*′ share also common characteristics. That is, if two terms have the same “is-a” depth, they share common characteristics. On the other hand, consider that term *A* “part-of” term *C* and term *B* “part-of” term *C*. We may not be able to infer common characteristics between *A* and *B*. Therefore, we use “is-a” relation and not “part-of” (or other relations) for computing the depth of terms, because the depth (specificity) of a GO term *t* based on its “is-a” *relations* influences the semantic relationships of *t* with the other terms that have no hierarchical relationships with *t*. *Thus, the depth of a term node is the number of “is-a” relations that connect it with the root term node (its “is-a” distance to the root).*

For example, recall Figure 1. The root term *biological process* (GO:0008150) has its own depth. Since both of the terms *multicellular organismal process* (GO:0032501) and *developmental process* (GO:0032502) inherit the same characteristics from their supertype GO:0008150, they both have the same depth. Alternatively, we can determine that these terms have the same depth, because they have the same *distance to the root* based on their *is-a* relations. As another example, the terms *kidney development* (GO:0001822), *system development* (GO:0048731), *multicellular organismal development* (GO:0007275), and *anatomical structure morphogenesis* (GO:0009653) have the same “is-a” depth and also common characteristics.

**Figure 4:**
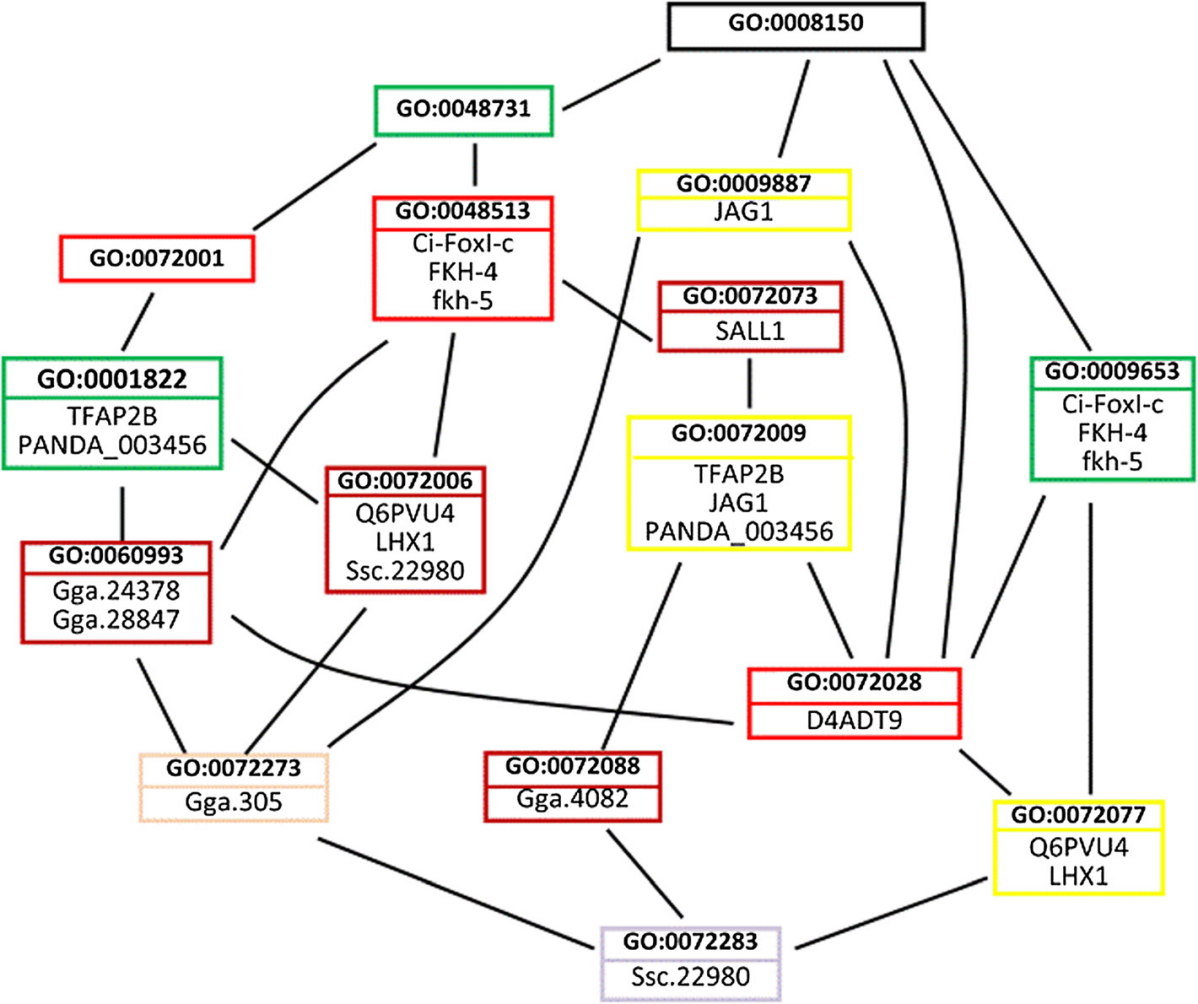
**The POG shown in Figure**3**after coloring each set of terms that have the same *depth *with the same color for easy reference.**

In the POG in Figure 4, each set of terms that have the same depth are colored with the same color for easy reference. For example, the terms *kidney development* (GO:0001822), *system development* (GO:0048731), and *anatomical structure morphogenesis* (GO:0009653) are colored with the same color as an indicative that they have the same depth. We note that the depths computed may not reveal equal conceptual depths for terms located in different subtrees in GO graph. For instance, in the example presented above, the terms *kidney development* and *system development* have the same depth even though the term *kidney development* is more specific than the term *system development*. This is because the two terms are located in two *different subtrees* in GO graph. If a term has multiple “is-a” inheritances, only its longest “is-a” distance to the root is considered.

**Figure 5:**
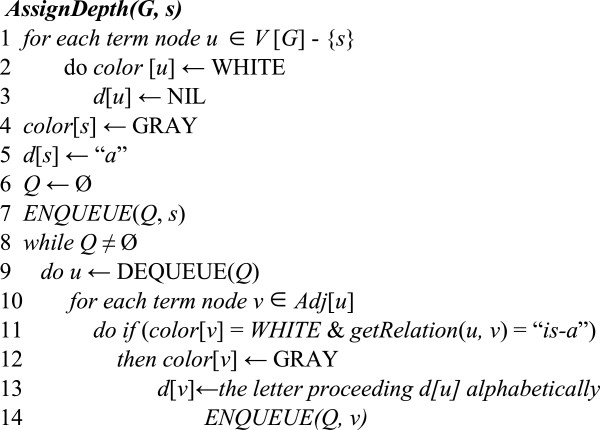
**Algorithm *AssignDepth.***

We constructed an algorithm called *AssignDepth* (see Figure 5) that determines the depths of GO terms. It employs *breadth-first search* techniques. The input to the algorithm is GO Graph *G* = (*V*, *E*), where *V* is the set of term and *E* is the set of edges representing the relations between the terms. *G* is represented by its adjacency list. The algorithm assigns an alphabetical letter to each hierarchical level in the graph based on “is-a” relations to denote the depth of the terms in this level. It starts at the root *s*, which is at level 0. In the first stage, it visits all the terms that are at the distance of one “is-a” edge away. Then, it visits all the new terms that can be reached at the distance of two “is-a” edges away from root term *s*. This process continues until every term has been visited. To keep track of progress, the algorithm *colors* each node either white or gray. A node is *discovered* the first time it is encountered during the search, at which time it becomes GRAY. The color of each node *u* ∈*V* is stored in variable *color*[*u*]. The algorithm uses a queue *Q* to manage the set of gray nodes. In line 11, function *getRelation*(*u, v*) returns the relation between two input terms *u* and *v*. Each term *v* is assigned an alphabetical letter that depicts its depth, which is stored in variable *d*[*v*].

### Determining the meaningful lowest common ancestors and the meaningful top common descendants of KCs

We observe that in order for a Lowest Common Ancestor (LCA) to be meaningful and in order for a Top Common Descendant (TCD) to be meaningful: (1) the LCA/TCD in POG should have a different *depth* than the KC, and (2) the path from the LCA/TCD to the KC in the POG should not include two or more terms with the same *depth*.

For example, consider Figure 4 and the query *Q*(“Gga.4082”, “LHX1”). The KC annotating the gene “Gga.4082” is *nephron epithelium morphogenesis* (GO:0072088) and the KCs annotating the gene “LHX1” are *nephron development* (GO:0072006), and *renal vesicle morphogenesis* (GO:0072077). One of the LCAs of the two KCs GO:0072088 and GO:0072077 is the term *nephron epithelium development* (GO:0072009). However, GO:0072009 is *not a meaningful* LCA for GO:0072077 *(i.e., GO:0072009*∉ *SR*_*GO:0072077*_*)*, because its depth is the same as that of GO:0072077 (recall Figure 4). Based on these observations, we now introduce proposition 1.

*Proposition 1: Meaningful LCA/TCD; A LCA/TCD is considered meaningful, if: (1) the* depth *of the LCA/TCD is different than the* depths *of the KCs, and (2) the path in the POG from the LCA/TCD to each of the KCs does not include two or more terms with the same* depth*.*

*Notation 2, DPT*_*x*_*; DPT*_*x*_*denotes the depth of GO term x.*

We prove observation/proposition 1 heuristically as follows. First, we prove: *if a LCA/TCDSR*_*KC*_*, then DPT*_*LCA/TCD*_*≠ DPT*_*KC*_. That is, in order for a LCA/TCD to be meaningful, its depth should be different than the depth of the KC. We are going to validate this observation by checking whether it conforms to the structural characteristics of *existence dependency*. The concept of existence dependency was first proposed for Entity-Relationship modelling [17]. An object *x* is *existence-dependent* on an object *y* if the existence of *x* is dependent on the existence of *y*[18]. The existence dependency concept and the SR_KC_ concept have correspondences: both denote that *an object(s) has a strong association with another object*. SR_KC_ is a set of GO terms, whose *existence* in POG is *dependent* on the existence of the KC *(or conversely, the existence of the KC in the graph is dependent on the existence of the set of terms)*. Snoeck et al. [16] argue that the existence dependency relation is a partial ordering of *object types (i.e., depths)*. The authors transform an OO schema into a graph consisting of the *object types* found in the schema and their relations. The object types in the graph are related only through associations that express existence dependency. The authors demonstrated through the graph that *an object type is never existence-dependent on itself.* That is, if the two objects *O*_*i*_ and *O*_*j*_ belong to the same type, *O*_*i*_ cannot be dependent on *O*_*j*_ and vice versa. This finding is in agreement with our proposed rule, when we view: (1) a GO term in GO Graph as an object, and (2) the GO term’s depth as the type of the object. Thus, if a LCA/TCD has the same depth as the KC, the LCA/TCD can never be existence-dependent on the KC (and vice versa); therefore, this LCA/TCD is meaningless and the genes annotated to it may not be semantically related to the genes annotated to the KCs.

Second, we prove: *If a LCA/TCD is semantically related to the KC, then DPT*_*Tx*_*DPT*_*Ty*_*where T*_*x*_*and T*_*y*_*are term nodes located between the LCA/TCD and the KC in POG*. We can verify this rule as follows. In order for LCA/TCD ∈SR_KC_, all term nodes located between the LCA/TCD and the KC in the POG have to be related to the KC. Let: (1) term *T*_*y*_∈SR_KC_, (2) *T*_*y*_ be a descendant of the KC, and (3) term *T*_*x*_ be a descendant of *T*_*y*_. In order for *T*_*x*_ to be semantically related to the KC, intuitively *T*_*x*_ has to be semantically related *T*_*y*_, because *T*_*y*_ relates (connects) *T*_*x*_ with the KC. If *T*_*x*_ and *T*_*y*_ have the same depth, then *T*_*x*_$\notin \;SR_{T_{y}}$ (according to the first rule). Therefore, in order for *T*_*x*_ to be semantically related to the KC, *DPT*_*Tx*_*DPT*_*Ty*_.

**Figure 6:**
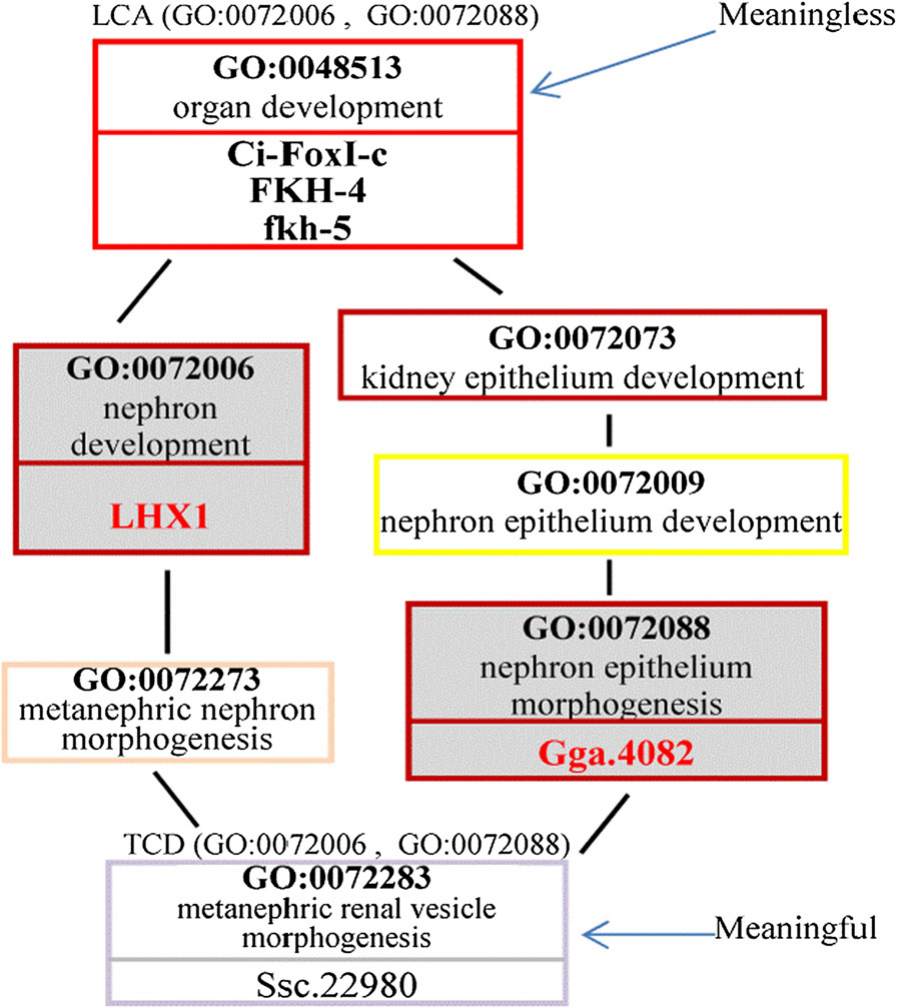
**GO:0048513 is a meaningless LCA of GO:0072006 and GO:0072088.** GO:0072283 is a meaningful TCD of GO:0072006 and GO:0072088.

**Figure 7:**
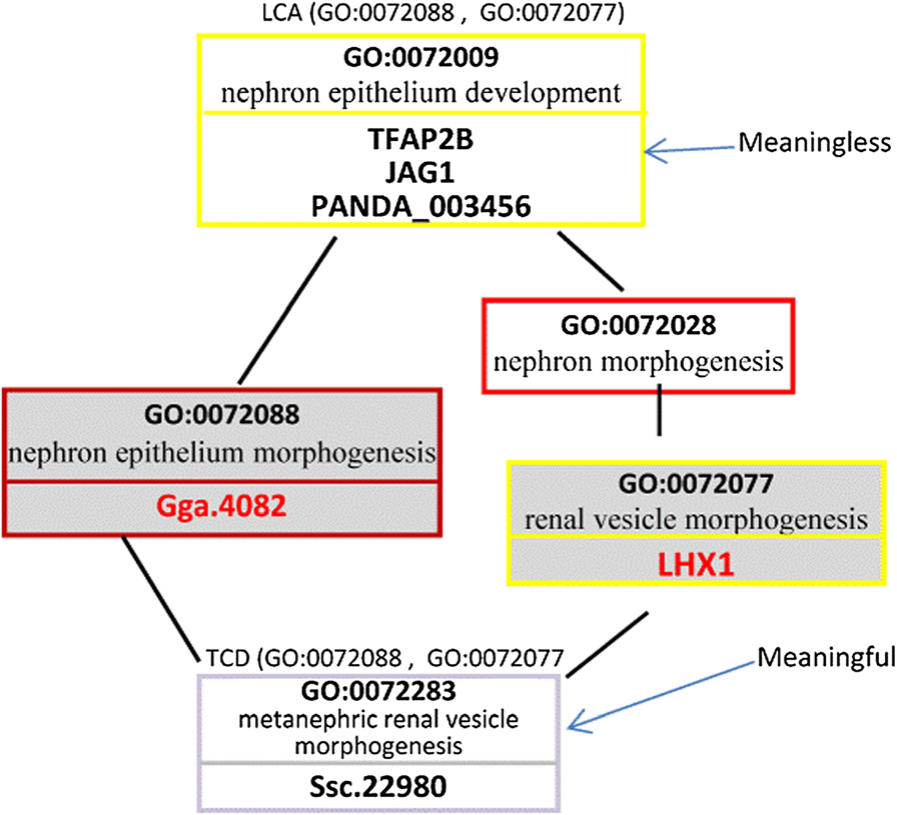
**GO:0072009 is a meaningless LCA of GO:0072088 and GO:0072077.** GO:0072283 is a meaningful TCD of GO:0072088 and GO:0072077.

*Example 1:* Consider Figure 4 and the query *Q*(“LHX1”, “Gga,4082”). As shown in Figure 4: (1) the KCs annotating the gene “LHX1” are *nephron development* (GO:0072006) and *renal vesicle morphogenesis* (GO:0072077), and (2) the KC annotating the gene “Gga.4082” is *nephron epithelium morphogenesis* (GO:0072088). As shown in Figure 6, one of the LCA of the two KCs is the term *organ development* (GO:0048513). However, by applying proposition 1, this LCA is meaningless, because the path in the POG from the LCA to the KC (GO:0072088) includes two terms with the same depth (i.e., the terms GO:0072073 and GO:0072088). Also, as shown in Figure 7, the term *nephron epithelium development* (GO:0072009) is a meaningless LCA of the two KCs, because this LCA and the KC (GO:0072077) have the same depth. Therefore, the genes annotated to these two meaningless LCAs *(i.e., the genes* “Ci-FoxI-c”, “FKH-4”, “fkh-5”, “*TFAP2B”, “JAG1”, and “PANDA_003456”)* will *not* be returned as the answer for the query *Q*(“Gga.4082”, “LHX1”). By applying proposition 1, the term *metanephric renal vesicle morphogenesis* (GO:0072283) is a meaningful TCD of the KCs (see Figures 6 and 7). Therefore, the gene annotated to GO:0072283 (i.e., the gene Ssc.22980) will be returned to the user as the answer for the query.

**Figure 8:**
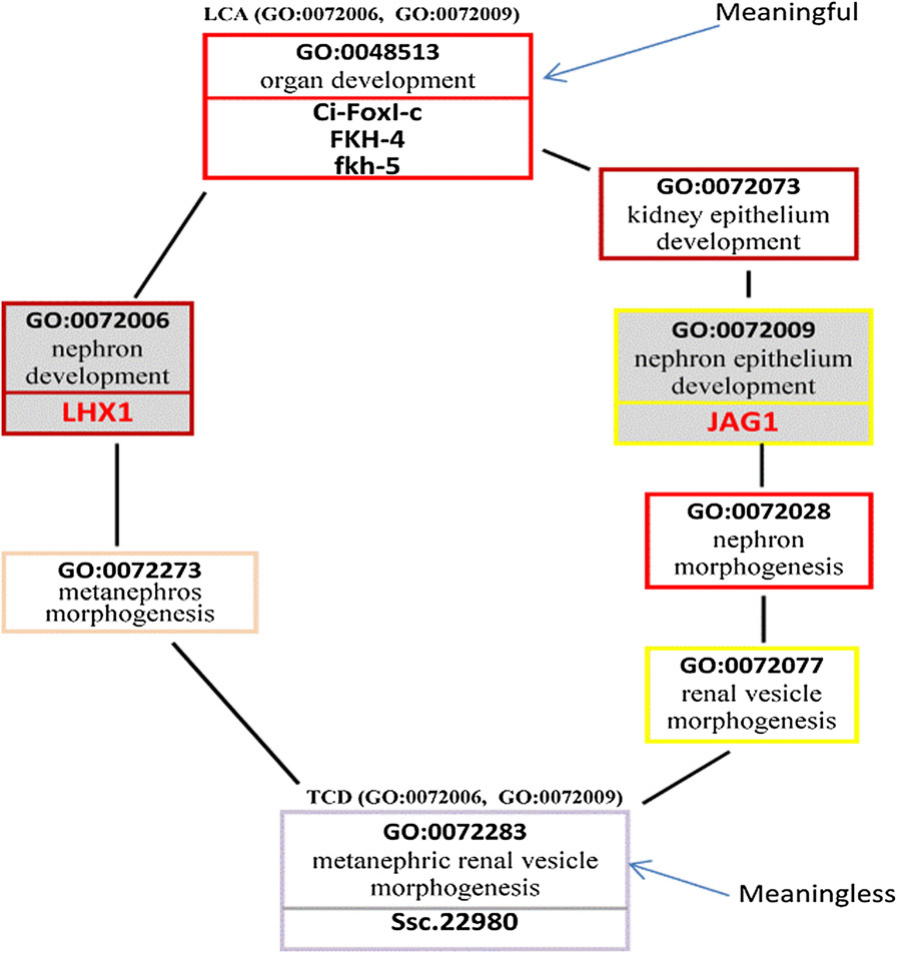
**GO:0048513 is a meaningful LCA of GO:0072006 and GO:GO:0072009.** GO:0072283 is a meaningless TCD of GO:0072006 and GO:GO:0072009.

*Example 2:* Consider Figure 4 and the query *Q*(“JAG1”, “LHX1”). By applying proposition 1 and as demonstrated by Figure 8, the term *organ development* (GO:0048513) is a meaningful LCA for the KCs *nephron xdevelopment* (GO:0072006) and *nephron epithelium development* (GO:0072009). Therefore, the genes annotated to GO:0048513 (*i.e., the genes “Ci-FoxI-c”, “FKH-4”, and “fkh-5”)* are semantically related to *both* of the input genes “LHX1” and “JAG1”. Therefore, these genes will be returned as the answer for the query. By applying proposition 1, the term *metanephric renal vesicle morphogenesis* (GO:0072283) is a meaningless TCD for the KCs, because the path from GO:0072283 to the KC (GO:0072009) includes two terms with the same depth (i.e., the terms GO:0072077 and GO:0072009). Therefore, the gene annotated to GO:0072283 (i.e., the gene Ssc.22980) will *not* be returned to the user as an answer for the query.

**Figure 9:**
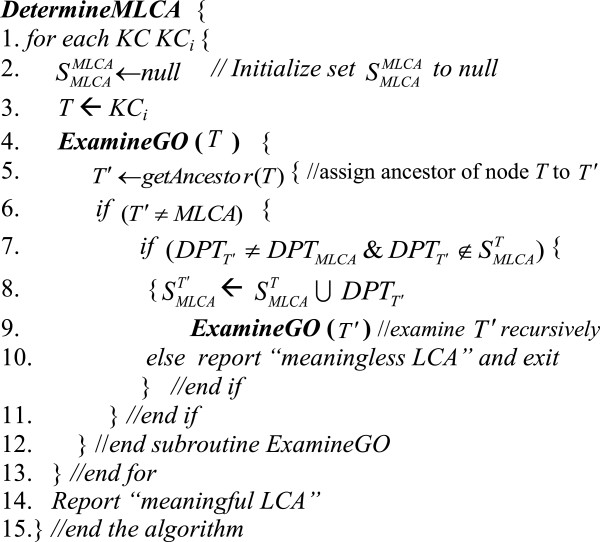
**Algorithm *DetermineMLCA.***

We constructed an algorithm called *DetermineMLCA* (see Figure 9) that checks whether an input LCA of KCs is *meaningful*. The input to the algorithm is a set of KCs and their LCA. For each KC in the set, lines 4–9 examine recursively each term *T*′ located between the KC and the LCA to check if its depth is different than the depth of the LCA and also different than the depths of all terms located between *T*′ and the KC. If the depths of terms between *each* KC and the LCA are distinct, line 14 will report that the LCA is a meaningful LCA.

We also constructed an algorithm called *DetermineMTCD* that checks whether an input TCD of KCs is *meaningful*. The algorithm is similar to algorithm *DetermineMLCA* in Figure 9, except that it examines the descendants of KCs rather than their ancestors.

### Ranking meaningful LCAs and meaningful TCDs

Each gene keyword of a query can be annotated to more than one GO term. Therefore, there may be more than one meaningful LCA/TCD for the query’s KCs. The degree of relativity of these LCAs/TCDs to the KCs may vary. Accordingly, the relativity of the genes described by these LCAs/TCDs to the input genes may vary. Therefore, we need a mechanism that ranks these LCAs/TCDs. We propose below techniques for ranking the LCAs/TCDs.

### Ranking with respect to one input gene keyword

Let *LCA*_*i*_ denote that GO term *i* is a LCA for a query’s KCs; let *R*(*LCA*_*i*_)denote the *rank* of*LCA*_*i*_. Let *KC*_1_denote the KC annotating input gene *g*_*1*_. Intuitively, the rank of *LCA*_i_ (i.e., *R*(*LCA*_*i*_)) with respect to *KC*_*1*_ is *R*(*KC*_1_) scaled down appropriately to account for the specificity of *LCA*_*i*_. We scale down the rank *R*(*KC*_1_) by a factor *decay*^*j*−1^ for each hierarchical level located between *KC*_*1*_ and *LCA*_*i*_ in GO Graph. *decay* is a parameter that can be set to a value in the range 0 to 1 (in our experiments we set decay to 0.7). “*j”* is the number of hierarchical levels between *KC*_*1*_ and *LCA*_*i*_. Let *R*(*LCA*_*i*_, *KC*_1_) denote the rank of *LCA*_*i*_ with respect to *KC*_*1*_. *R*(*LCA*_*i*_, *KC*_1_) is computed as follows:

(1) $$R\left(\mathrm{LC}A_{i},KC_{1}\right)=R\left(KC_{1}\right)\times \mathrm{deca}y^{j-1}$$

In the discussion above and in equation 1, we implicitly assumed that there is only one GO term annotating the gene *g*_*1*_. In case *g*_*1*_ is annotated to *n* GO terms, we compute the rank of each term using equation 1. Let the computed ranks be *r*_*1*_, *r*_*2*_, …, *r*_*n*_. The combined rank is:

(2) $$\hat{R}\left(\mathrm{LC}A_{i},KC_{1}\right)=f\left(R_{1},R_{2},\ldots ,R_{n}\right)$$

Where *f* is some aggregation function. We set *f = max* by default, but other choices (such as *f = sum*) are also possible.

### Overall ranking

Equations 1 and 2 show the rank of *LCA*_*i*_ with respect to only one gene keyword. In case there are *m* gene keywords in the query (i.e., *Q(“g*_*1*_*”, “g*_*2*_*”, …, “g*_*m*_*” )*, the overall ranking is the rank of *LCA*_*i*_ with respect to the *m* KCs annotating these genes. That is, the overall ranking of *LCA*_*i*_ for query *Q(“g*_*1*_*”, “g*_*2*_*”, …, “g*_*m*_*” )* is computed as shown in equation 3.

(3) $$\begin{array}{l} R\left(\mathrm{LC}A_{i}\right)=\left(\sum_{1\leq j\leq m}\hat{R}\left(\mathrm{LC}A_{i},KC_{j}\right)\right)\\ \;\times \mathrm{prox}\left(\mathrm{LC}A_{i},\left(g_{1},g_{2},\ldots ,g_{m}\right)\right)\; \end{array}$$

The overall ranking is the sum of the ranks with respect to each gene keyword in the query, multiplied by a measure of keyword proximity: *prox*(*LCA*_*i*_, (*g*_1_, *g*_2_. …, *g*_*m*_)). The keyword proximity function *prox*(*LCA*_*i*_, (*g*_1_, *g*_2_, …, *g*_*m*_)) can be any function that ranges from 0 *(gene keywords are annotated to terms that are very far apart in GO Graph)* to 1 *(gene keywords are annotated to terms that occur right next to each other in GO Graph)*.

By default, we set our proximity function to be inversely proportional to the size of the smallest text window in *LCA*_*i*_ containing the occurrences of all the input gene keywords *g*_*1*_*, g*_*2*_*, …, g*_*m*_. The smallest text window is the rectangle in GO graph that contains the relevant occurrences of all input gene keywords. The height of the window/rectangle is the number of hierarchical levels between the highest and lowest GO terms annotating the input gene keywords. The width of the window/rectangle is the number of GO terms between the right-most and left-most terms annotating the input gene keywords.

### ***Computing****R*(*KC*_*j*_)

The algorithm for computing *PageRank*[19] of HTML documents employs the following formula:

(4) $$p\left(v\right)=\frac{1-d}{N_{d}}+d\times \sum_{\left(u,v\right)\in \mathrm{HE}}\frac{p\left(u\right)}{N_{h}\left(u\right)}$$

•*p(v)*: the *PageRank* of a document *v*

•*N*_*d*_: the total number of documents.

•*N*_*h*_*(v)*: the number of out-going hyperlinks from document *v*.

•*d* is a parameter usually set to 0.85.

•*HE:* hyperlink edge*.*

As the formula above shows, *PageRank* is a sum of two probabilities. The first is *(*1*-d)/N*_*d*_, which is the probability of visiting document *v* at random. The second is the probability of visiting document *v* by navigating through other documents, which is calculated by the sum of the normalized *PageRank*s of all documents that connect to *v* by hyperlinks, multiplied the probability of navigation *d*.

We are going to adapt the *PageRank* formula to GO Graphs by mapping GO terms to documents and hyperlink edges to edges connecting the GO terms. As there is a tight coupling between each two Web pages connected by a hyperlink, there is a tight coupling between each two GO term nodes connected by a containment edge. If term node *u* is important (i.e., has high score), it is likely that its children and parents are important too. If the children and parents of *u* are important (i.e., have high scores), it is likely that *u* is important too. By performing mapping, adaptation, and adjustments to the *PageRank* formula, we constructed the following formula for computing *R*(*KC*_*j*_):

(5) $$R\left(KC_{j}\right)=\left(1-p_{1}-p_{2}\right)S\left(KC_{j}\right)+p_{1}\sum \frac{S\left(v\right)}{\mathrm{Ni}\left(v\right)}+p_{2}\sum \frac{S\left(v\right)}{\mathrm{Ni}\left(v\right)}$$

(6) $$S\left(v\right)=\sum_{i=0}^{k-1}\frac{\left(\begin{array}{c} b\\ i \end{array}\right)\left(\begin{array}{c} a-b\\ c-i \end{array}\right)}{\left(\begin{array}{c} a\\ c \end{array}\right)}$$

•*v*: The parent(s) of *KC*_*j*_

•*p*_*1*_: A discretionary parameter that denotes the probability of navigating from *KC*_*j*_through an edge to term *v*, because the gene the user is looking for is better described by *v*. In our experiments we set *p*_*1*_ to 0.4.

•*p*_*2*_: A discretionary parameter that denotes the probability of navigating from term *v* through an edge to *KC*_*j*_, because the gene the user is looking for is better described by *KC*_*j*_. In our experiments we set *p*_*2*_ to 0.6.

•*Ni*(*v*): The number of edges entering node *v*.

•*S*(*v*) and *S*(*KC*_*j*_): The scores of terms *v* and *KC*_*j*_ respectively, and are computed as follows. Let: (1) “*a”* be the set of genes in microarray, (2) “*b”* be the set of genes annotated to term *v* (or to *KC*_*j*_), and (3) “*c”* be the number of significant genes in microarray. The score *S*(*v*) is the probability that the number of significant genes annotated to term *v* is exactly “*k”* out of the “*c*” significant genes^a^, and it is given by the following Fisher’s exact test [20]:

To compute *S*(*v*), GRank provides reference sets of microarrays. Example 3, provides an example illustrating the calculation of *S*(*v*).

*Example 3:* Consider the microarray titled “*GeneChip Human Genome U133A 2.0 Array*”, which represents 14500 unique human genes and 417 significant unique genes. Let us compute the probability that the number of significant genes annotated to the term *anatomical structure development* (GO:0048856) is exactly 300 out of the 417 significant genes of the microarray. That is, we want to compute the score *S*(GO:0048856). There are 10153 unique genes annotated to GO:0048856. Therefore, the probability is computed as shown in equation 7:

(7) $$\begin{array}{l} S\left(\mathrm{GO}:0048856\right)=\sum_{i=0}^{299}\frac{\left(\begin{array}{c} 10153\\ i \end{array}\right)\left(\begin{array}{c} 14500-10153\\ 417-i \end{array}\right)}{\left(\begin{array}{c} 14500\\ 7 \end{array}\right)}\\ \;=0.73\; \end{array}$$

## Results and discussion

We experimentally evaluated the quality of GRank and compared it with DynGO [1]. DynGO “retrieves genes and gene products that are *relatives* of input genes based on similar GO annotations, and displays the related genes and gene products in an association tree” [1,2]. DynGO “can support heavier computations and supports semantic retrieval of both similar terms and gene products” [2]. We implemented GRank in Java, run on Intel(R) Core(TM)2 Duo CPU processor, with a CPU of 2.6 GHz and 4 GB of RAM, under Windows 7.

### Benchmarking datasets

Pathways are sets of genes shown to have high functional similarity and can be used to validate similarity measures [7,14,21]. A fully described pathway represents the dynamics and dependencies among a set of gene/gene products. Therefore, we used in our experiments pathways as a reference for evaluating and comparing the similarity measures/relationships of GRank and of DynGO [1]. Given a set *S* of genes, the methods should return another set *S*′ of genes that are semantically related to *S*. In order for sets *S* and *S*′ to be related, *S* and *S*′ should be part of a *same pathway*.

We used for the evaluation two different benchmarks: KEGG and Pfam benchmarks. We selected a set of 15 human and 15 yeast diverse KEGG pathways (see Tables 2 and 3); the genes were retrieved using the DBGET database [22]. We selected 15 groups of highly related Pfam entries (see Table 4) from the Sanger Pfam database [23]. The percentage of non-IEA annotations is different in the yeast and human. It is about 70% for the yeast annotations compared to about 60% for the human annotations. That is, there is a higher contribution of non-IEA annotation in yeast than in human.

**Table 2:** The 15 KEGG Human Pathways used in the experiments

**Class**	**Human**		
	**Pathway**	**Name**	**# of genes**
Carbohydrate Metabolism	hsa00040	Pentose and glucuronate interconversions	34
Energy Metabolism	hsa00920	Sulfur metabolism	14
Lipid Metabolism	hsa00140	Steroid hormone biosynthesis	26
Amino Acid Metabolism	hsa00290	Valine, leucine and isoleucine biosynthesis	5
Glycan Biosynthesis and Metabolism	hsa00563	Glycosylphosphatidylinositol	25
Metabolism of Cofactors and Vitamins	hsa00670	One carbon pool by folate	19
Biosynthesis of Other Secondary Metabolites	hsa00232	Caffeine metabolism	7
Transcription	hsa03022	Basal transcription factors	23
Folding, Sorting and Degradation	hsa04130	SNARE interactions in vesicular transport	36
Replication and Repair	hsa03450	Non-homologous end-joining	13
Replication and Repair	hsa03430	Mismatch repair	23
Fatty acid metabolism	Hsa00085	Fatty acid biosynthesis	12
Cellular Processes	hsa04950	Maturity onset diabetes of the young	25
Signal Transduction	hsa04803	Homo sapiens	16
Lipid Metabolism	hsa00120	Primary bile acid biosynthesis	14
**Total number of genes**			**292**

**Table 3:** The 15 KEGG Yeast Pathways used in the experiments

**Class**	**Yeast**		
	**Pathway**	**Name**	**# of genes**
Carbohydrate Metabolism	sce00562	Inositol phosphate metabolism	15
Energy Metabolism	sce00920	Sulfur metabolism	15
Lipid Metabolism	sce00600	Sphingolipid metabolism	13
Amino Acid Metabolism	sce00410	beta-Alanine metabolism	12
Glycan Biosynthesis and Metabolism	sce00514	Saccharomyces cerevisiae	13
Metabolism of Cofactors and Vitamins	sce00670	One carbon pool by folate	15
Biosynthesis of Other Secondary Metabolites	sce00903	Limonene and pinene degradation	20
Transcription	sce03022	Basal transcription factors	32
Folding, Sorting and Degradation	sce04130	SNARE interactions in vesicular transport	23
Replication and Repair	sce03450	Non-homologous end-joining	10
Replication and Repair	sce04070	Phosphatidylinositol signaling system	15
Fatty acid metabolism	sce04140	Regulation of autophagy	17
Cellular Processes	sce04111	Saccharomyces cerevisiae	25
Signal Transduction	sce04011	MAPK signaling pathway	57
Lipid Metabolism	sce03010	Ribosome	12
**Total number of genes**			**294**

**Table 4:** The15 Pfam Human Clans and the 15 Pfam Yeast Clans used in the experiments

**Pfam accession**	**Pfam ID**	**Number of genes (human)**	**Number of genes (yeast)**
CL0406	vWA-like	11	6
CL0344	4Fe-4S	7	4
CL0461	Metallothionein	18	11
CL0020	TPR	13	6
CL0418	GIY-YIG	8	19
CL0417	BIR-like	10	6
CL0233	SufE_NifU	9	10
CL0167	Zn_Beta_Ribbon	7	5
CL0099	ALDH-like	18	11
CL0042	Flavoprotein	10	7
CL0040	tRNA_synt_II	12	2
CL0179	ATP-grasp	7	6
CL0417	BIR-like	11	9
CL0445	SNARE-fusion	8	6
CL0444	YNI	9	5
**Total number of genes**		**158**	**113**

For each group, we retrieved the corresponding human and yeast gene identifiers from the Uniprot database [24]. Assuming that genes belonging to the same KEGG pathway are often related to a similar biological process (BP), the similarity values calculated for this dataset should be related to the BP aspect. Also, genes sharing common domains in a Pfam clan often have a similar molecular function (MF), the similarity values calculated for this second dataset should be related to the MF aspect.

### Evaluating recall and precision

We measured the *recall (or true positive rate)* and *precision* of GRank and of DynGO [1]. Let: (1) *G*_*P*_ be all genes in a pathway and *n* be the number of these genes, and (2) *G*_*M*_ be the *m* genes retrieved by one of the methods as semantically related to the input gene keywords:

(8) $$\mathrm{Recall}=\;\left(\left|G_{M}\;\cap G_{P}\right|/n\right)$$

(9) $$\mathrm{Precision}=\left(\left|G_{M}\cap G_{P}\right|/m\right)$$

**Figure 10:**
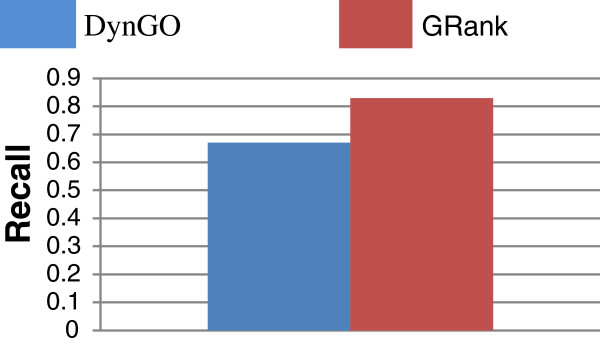
Overall average recall for DynGO and GRank.

**Figure 11:**
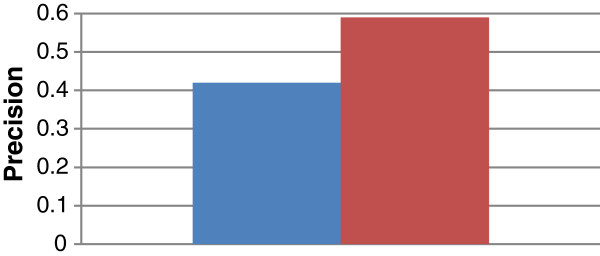
Overall average precision for DynGO and GRank.

**Figure 12:**
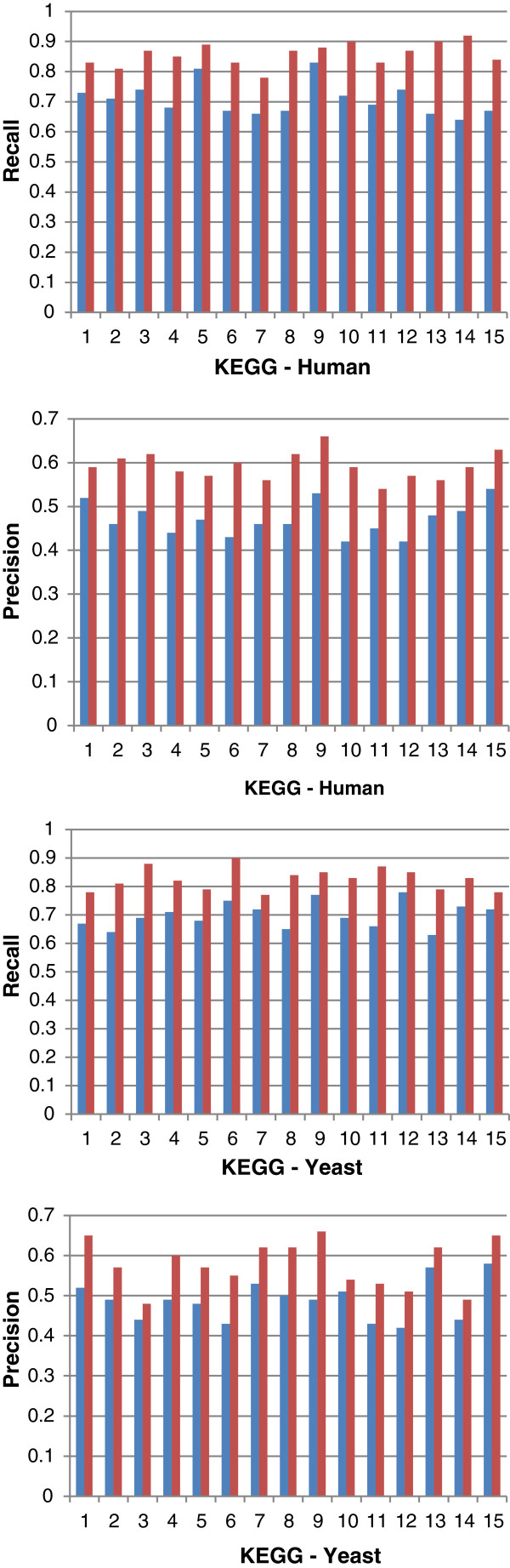
Recall and precision of DynGO and GRank obtained with the 15 human and 15 yeast diverse KEGG pathways.

**Figure 13:**
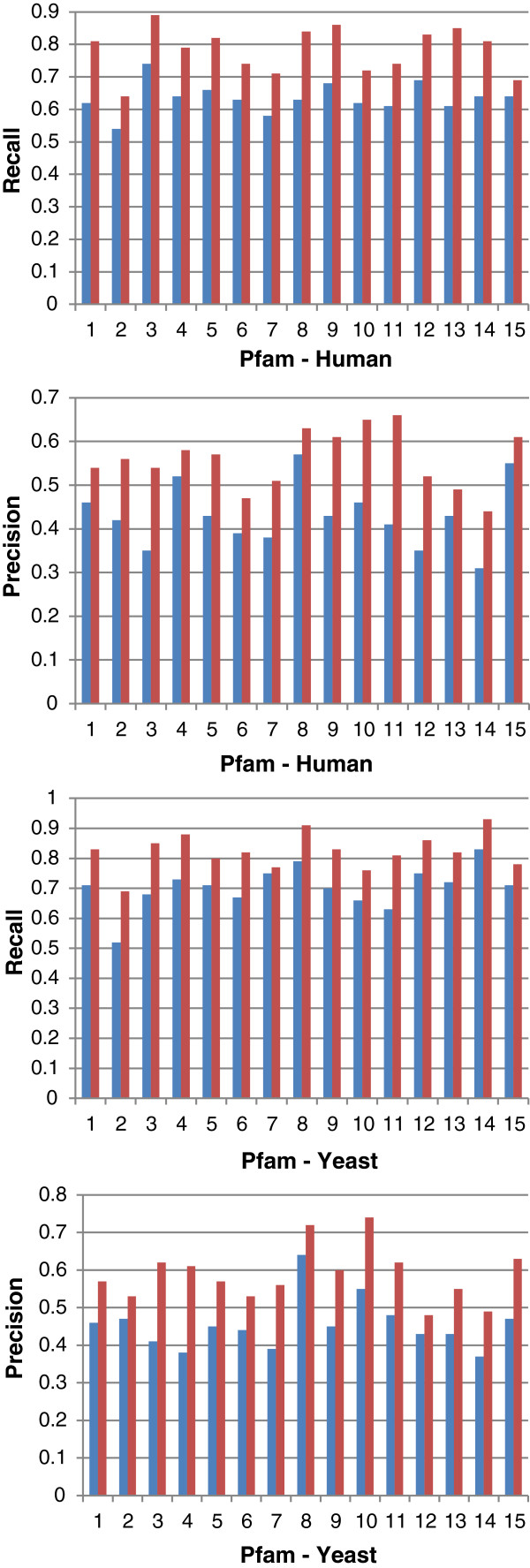
Recall and precision of DynGO and GRank obtained with the 15 human and 15 yeast diverse Pfam clans.

Figures 10 and 11 show the *overall average* recall and precision respectively for DynGO and GRank using the 857 genes of the two benchmarks. Figure 12 shows the recall and precision results obtained with the KEGG pathways. Figure 13 shows the recall and precision results obtained with the pfam clans. For each KEGG and pfam pathway/clan (x-axis), the recall and precision values are represented as histograms (y-axis).

As the figures show, recall and precision values vary based on: (1) pathways, and (2) the accuracy of each of the two methods to capture the semantic similarities and relationships among gene annotations within pathways. We can conclude that the recall and precision values for the two benchmarking datasets showed that GRank significantly outperforms DynGO. The results reveal the robustness of the GRank’s method and its ability to reflect the semantic relationships among gene annotations.

Table 5 shows the proportion of the meaningful LCAs versus the meaningful TCDs in the different sets of the benchmarks, where the significant answer genes are annotated to.

**Table 5:** The proportion of the meaningful LCAs versus the meaningful TCDs in the different sets of the benchmarks

***Pathway***	***Percentage of the significant genes found at the meaningful LCAs***	***Percentage of the significant genes found at the meaningful TCDs***
KEGG Human	67%	33%
KEGG Yeast	74%	26%
Pfam Human	72%	28%
Pfam Yeast	86%	14%

To further analyze the behavior of the two methods, we classified GO Graphs into six criteria based on the relative position of a KC in the graph and on the type of relations that connect the KC with other term nodes *(the classification is shown in* Table 6*)*. We analyzed the behavior of GRank and of DynGO in terms of their *recall* and *precision* under each of the six criteria. We computed the average recall and precision of the two methods under *each of the six criteria*. The results are shown in Table 7.

**Table 6:** **Classification of GO Graphs *(C# denotes criterion number)***

***C#***	***GO Graph criterion***
***1***	All KCs in the GO Graph connect to their ancestor GO term nodes by “is-a” relations *only*
***2***	All KCs in the GO Graph connect to their ancestor GO term nodes by “part-of” relations *only*
***3***	KCs in the GO Graph connect to their ancestor GO term nodes by both, “is-a” and “part-of” relations.
***4***	KCs are in shallow hierarchical levels in the GO Graph. *We consider a hierarchical level is shallow if it is less than six*
***5***	KCs are in deep hierarchical levels in the GO Graph
***6***	Some KCs are in deep hierarchical levels in the GO Graph and others are in shallow levels

**Table 7:** Average Recall and Precision of GRank and DynGO under the Six Criteria

**C#**	**DynGO**		**GRank**	
	***Recall***	***Precision***	***Recall***	***Precision***
**1**	0.54	0.34	0.44	0.14
**2**	0.85	0.64	0.94	0.80
**3**	0.68	0.58	0.81	0.67
**4**	0.63	0.25	0.89	0.66
**5**	0.80	0.52	0.89	0.66
**6**	0.46	0.23	0.89	0.69

As Table 7 shows:

(1) GRank outperforms DynGO under criteria 2–6.

(2) GRank does not perform well under criterion 1. More research work needs to be conducted to overcome the shortcoming of this criterion.

(3) GRank achieved the same recall values and the same precision values under criteria 4, 5, and 6, which is an indicative that the locations of KCs in GO Graph are *irrelevant* to the performance of GRank and that its performance does not vary with the height of a GO Graph.

GRank performance under criteria 2–6 is due to: (1) its consideration to the *structural dependencies* among annotation terms and to its term-depth consideration, and (2) the fact that each of these criteria requires a method to account for the structural dependencies among annotation terms. The extent of the importance of the structural dependencies among annotation terms to a criterion differs from criterion to another: *it is more important to criterion 6 than to the other five criteria, which explains the substantial performance of GRank over DynGO methods under criterion 6.*

### Evaluating the impact of disregarding the ranking of meaningful LCA and TCD

**Figure 14:**
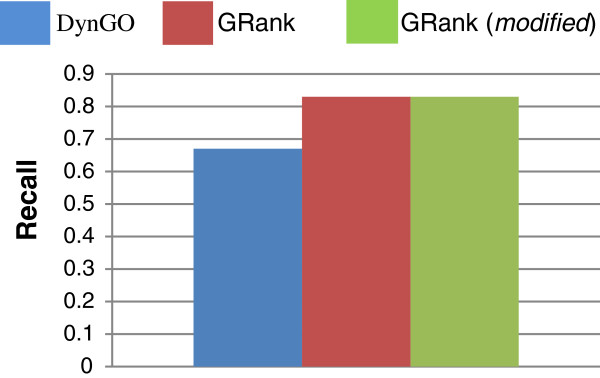
Average recall of the modified copy of GRank and of the original GRank and DynGO.

**Figure 15:**
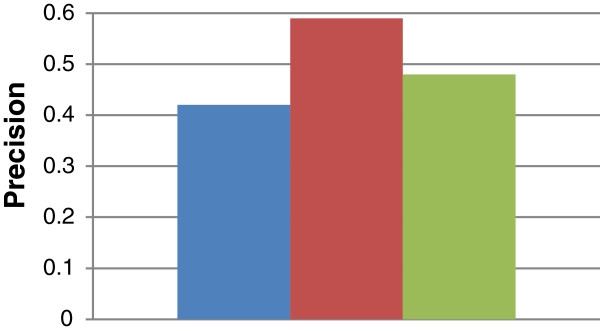
Average precision of the modified copy of GRank and of the original GRank and DynGO.

We modified a copy of GRank by *removing* its capability to rank meaningful LCAs and TCDs. Our objective is to study the impact of overlooking the ranking of meaningful LCAs and TCDs on the search quality of GRank. We aim at: (1) measuring the *decline* in GRank’s recall and precision as a result of disregarding ranking, and (2) comparing the modified copy’s recall and precision with DynGO. If the modified copy outperforms the DynGO method, this performance would be attributed to only GRank’s computation of meaningful LCAs and TCDs. Figures 14 and 15 show the overall average recall and precision respectively of the modified copy using the 857 genes of the two benchmarks *(for ease of comparison, we show also in the figure the average recall and precision of the original version of GRank and of DynGO).*

As Figure 14 shows, the overall average recall has not changed, which is expected, since ranking does not have an impact on recall; rather, it has an impact on precision. As the Figure 15 shows, the overall average *precision* of the modified copy is:

(1) Less than that of the original copy by 26%.

(2) Higher than that of DynGO.

We can conclude that the concept of meaningful LCA and TCD has significant impact on GRank’s search quality, while ranking has important but not significant impact on GRank’s search quality.

### Evaluating an alternative approach to overcoming the shortcoming of criterion 1

**Figure 16:**
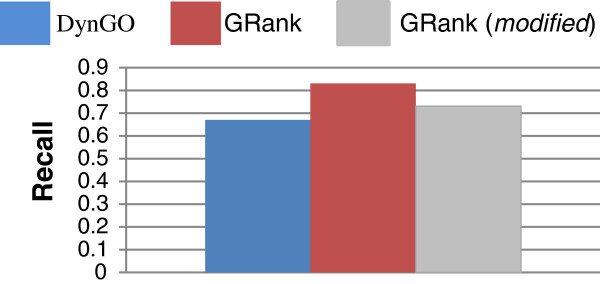
Average recall of the modified copy of GRank and of the original GRank and DynGO.

**Figure 17:**
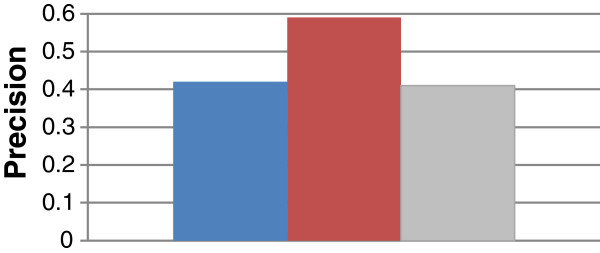
Average precision of the modified copy of GRank and of the original GRank and DynGO.

As discussed previously that GRank does not perform well under query criterion 1 *(recall* Tables 6 and 7*)* and that more research work needs to be done to overcome this limitation. However, our objective in this test is to evaluate a counter-technique, where a complete GO Graph is used instead of POG for computing meaningful LCAs and TCDs. Therefore, terms that connect with other terms via only is-a relations will also be considered in the computation of meaningful LCAs and TCDs. Towards this, we modified a copy of GRank so that it processes GO Graph instead of POG for computing meaningful LCAs and TCDs. Figures 16 and 17 show the average recall and precision of the modified copy using the 857 genes. As Figure 16 shows, the average recall of the modified copy is less than that of the original version by 15%, but is still higher than that of DynGO. However, as Figure 17 shows, the average precision of the modified copy is less than that of the original copy by 33%, and is slightly less than DynGO. We conclude that the concept of POG has a *significant* impact on GRank’s search quality and it compensates for the shortcoming of criterion 1.

## Conclusions

In this paper, we proposed a search engine called GRank that determines the semantic relationships among genes and gene products. GRank overcomes the limitations of current gene similarity measures by using the concept of *existence dependency* to determine the semantic relationships among gene annotations. It determines the *structural dependencies* among the GO terms annotating a given set *S* of genes in order to identify the set of other genes that are *semantically related* to the set *S*. Towards this, the framework of GRank refines the concept of LCA and TCD by introducing the concept of *meaningful* LCA and *meaningful* TCD. Given a set of genes *g*_*1*_, *g*_*2*_, …*g*_*n*_, GRank identifies the meaningful LCA and the meaningful TCD of the terms annotating *g*_*1*_, *g*_*2*_, …*g*_*n*_. The genes annotated to the meaningful LCA and the meaningful TCD have the closest semantic relationships with *g*_*1*_, *g*_*2*_, …*g*_*n*_. GRank ranks the meaningful LCAs and the meaningful TCDs based on their semantic relationships with KCs. We experimentally evaluated the quality of GRank and compared it with DynGO [1] using KEGG and Pfam benchmarks. In summary, the recall and precision values for the two benchmarking datasets showed that GRank outperforms DynGO. The experiments showed that GRank does not perform well if *all* KCs in GO Graph are connected to other term nodes with *only* “is-a” relations. We will investigate techniques in a future work that overcome this limitation.

## Endnote

^a^The same thing applies to the score S(*KC*_*j*_).

## Competing interests

The authors declare that they have no competing interests.

## Authors’ contributions

KT conceived, designed, and supervised the research. KT drafted and revised this manuscript. KT, HAM, DM, and ZAM performed the implementation of the research project. KT, HAM, DM, and ZAM carried out the experiments and the analysis of the results. All authors read and approved the final manuscript.
